# Social Network Behavior and Engagement Within a Smoking Cessation Facebook Page

**DOI:** 10.2196/jmir.5574

**Published:** 2016-08-02

**Authors:** Heather Cole-Lewis, Adler Perotte, Kasia Galica, Lindy Dreyer, Christopher Griffith, Mary Schwarz, Christopher Yun, Heather Patrick, Kisha Coa, Erik Augustson

**Affiliations:** ^1^ ICF International Rockville, MD United States; ^2^ Department of Biomedical Informatics Columbia University Medical Center New York, NY United States; ^3^ ICF International Washington, DC United States; ^4^ ICF International Fairfax, VA United States; ^5^ Applied Behavior Change Science Science and Product Envolve PeopleCare Bethesda, MD United States; ^6^ Tobacco Control Research Branch Division of Cancer Control and Population Sciences National Cancer Institute Rockville, MD United States

**Keywords:** social network analysis, smoking cessation, Facebook, social support, Web-based communities, social media, communication

## Abstract

**Background:**

Social media platforms are increasingly being used to support individuals in behavior change attempts, including smoking cessation. Examining the interactions of participants in health-related social media groups can help inform our understanding of how these groups can best be leveraged to facilitate behavior change.

**Objective:**

The aim of this study was to analyze patterns of participation, self-reported smoking cessation length, and interactions within the National Cancer Institutes’ Facebook community for smoking cessation support.

**Methods:**

Our sample consisted of approximately 4243 individuals who interacted (eg, posted, commented) on the public Smokefree Women Facebook page during the time of data collection. In Phase 1, social network visualizations and centrality measures were used to evaluate network structure and engagement. In Phase 2, an inductive, thematic qualitative content analysis was conducted with a subsample of 500 individuals, and correlational analysis was used to determine how participant engagement was associated with self-reported session length.

**Results:**

Between February 2013 and March 2014, there were 875 posts and 4088 comments from approximately 4243 participants. Social network visualizations revealed the moderator’s role in keeping the community together and distributing the most active participants. Correlation analyses suggest that engagement in the network was significantly inversely associated with cessation status (Spearman correlation coefficient = −0.14, *P*=.03, N=243). The content analysis of 1698 posts from 500 randomly selected participants identified the most frequent interactions in the community as providing support (43%, n=721) and announcing number of days smoke free (41%, n=689).

**Conclusions:**

These findings highlight the importance of the moderator for network engagement and provide helpful insights into the patterns and types of interactions participants are engaging in. This study adds knowledge of how the social network of a smoking cessation community behaves within the confines of a Facebook group.

## Introduction

Although the prevalence of smoking has been steadily declining, an estimated 42.1 million adults in the United States still currently smoke cigarettes [1,2]. Tobacco use continues to be the single largest preventable cause of death and disease in the United States, accounting for 1 of every 5 deaths [1,2]. Reducing the number of smoking individuals remains crucial for improving public health in the United States. For this reason, the National Cancer Institutes’ (NCIs) Smokefree.gov program exists as an open access Web-based smoking cessation intervention designed to provide resources and support to current smokers as well as family and friends looking to support someone who wants to quit smoking.

The Smokefree.gov program evolved from a single website to a suite of resources and supporting platforms as technology and literature for Web-based health interventions has advanced. There has been an emphasis on providing personalized resources for specific subgroups of smokers, and this analysis focuses on the Smokefree Women Facebook page, a resource developed specifically for women. Approximately 15% of women in the United States smoke [3], and female smokers face unique challenges in quitting, including weight concerns, quitting while pregnant, stress, and depression and thus can potentially benefit from gender-specific smoking cessation resources [4]. To this end, the Smokefree Women Facebook page was launched in 2009 to engage women and their social networks in the conversation on quitting smoking. The Smokefree Women Facebook page is meant to serve as a virtual support community and provides quit smoking and health information from the NCI to encourage members to lead healthier, smoke-free, lives and to engage women and their social networks in the conversation on quitting smoking. The purpose of the current analysis is to better understand the social network behavior and interactions of participants within the Smokefree Women Facebook group.

Systematic reviews of the literature on Web-based social networks for health indicate the need for further use and evaluation of tools such as Facebook for health-related information dissemination, surveillance, observation, prediction, and behavior change [5-7]. Maher et al [7] call for novel applications of computational methodologies to allow for nuanced understanding of social network sites without manipulation of the network. One such approach, social network analysis, is a collection of computational methods that can provide insight into the structure of a social networking site and interactions and behaviors among participants.

Existing research using social network analysis uncovers phenomena that may contribute to the efficacy of interventions. For example, in a controlled social network experiment, it was found that social reinforcement signals facilitated by clustering of social ties improved individual behavior adoption—adoption in this study being the act of registering for an Internet-based health forum [8]. Beyond benefits for individual action, clustered networks served to diffuse the behavior more quickly than random networks [6]. In addition, a social network analysis study on the smoking cessation website “QuitNet” concluded that characteristics for a sustainable social network include persistence of members over time, heterogeneity of cessation status, and bidirectional communications [9]. In a content-based network analysis of "QuitNet," theme-based thresholds (eg, support theme, advice theme) for identification of meaningful theme-based social subnetworks and identification of opinion leaders and subcommunity clusters within the theme-based networks [10]. Further application of social network analysis in existing behavior change support communities may uncover underlying psychosocial mechanisms useful for driving innovation and strategy in public health interventions.

To our knowledge, few if any studies have performed social network analysis within Facebook groups. Given the nature of the platform, network behavior in a Facebook group is inherently confounded by the Facebook algorithm [11]. In other words, all content, interactions, and users within and outside the group are subject to various manipulations that serve the goal of the host. Rather than deter the study of networks on Facebook, these conflicts mandate their study. Many health interventions, resources, programs, and naturally spawned social support groups live on Facebook due to its ease of usage and large existing audience. To support and improve the functionality of these communities, visualizations of network behavior within the confines of the platform are necessary.

Launched in 2009, the Smokefree Women Facebook group saw improved growth and engagement after a significant strategy shift documented by Post et al [12]. The strategy modification focused on repurposing user-generated content to encourage engagement in lieu of primarily promoting Smokefree resources [12]. With over 22,000 fans (“likes” on the page) at the time of data collection averaging 1700 user comments, 110 shares, and 6300 likes monthly, the community was ripe for an exploration of user interactions.

The goal of this study was to analyze patterns of participation, self-reported smoking cessation length, and interactions within the Smokefree Women Facebook group. In addition to visualizing the network structure, this study sought to analyze user content for potential themes and explore correlates of self-reported cessation length with placement within the network. The study combined quantitative and qualitative methods to answer the following exploratory research questions: (1) What are the characteristics of the network structure? and (2) How do people interact in the social networking site?

## Methods

### Sample

The Smokefree Women Facebook page is an open-access smoking cessation community. Built as an extension of the NCIs’ Web-assisted tobacco cessation intervention, Smokefree.gov, the Smokefree Women Facebook page was specifically created for the purpose of helping individuals achieve sustained abstinence from smoking. On the Smokefree Women Facebook page, participants communicate and interact with one another and the moderator by likes, comments, comment likes, and shares (see Table 1 for description). The page moderator (“Page Admin” or “Smokefree Women”), a trained public health professional, frequently posts unique and participant-generated content to the page to facilitate engagement, share information, and support women in smoking cessation. At the time of data collection, the Smokefree Women Facebook page had over 22,000 fans, or participants who had “liked” the page, with up to 2500 participants actively interacting on the Smokefree Women Facebook page monthly. Our sample consisted of individuals who interacted (eg, posted, commented) on the Smokefree Women Facebook page between February 2013 and March 2014 (n=4243).

**Table 1:** Facebook interactions and definitions.

Interaction	Definition
Moderator posts	Content posted by the moderator on the Facebook page wall (visible to all participants who visit the page and visible on “Home” newsfeed of participants based on Facebook algorithm)
Participant posts	Content posted by participants to the Smokefree Women Facebook page wall (visible on the left side of the page to all participants who visit the page but not necessarily visible on “Home” newsfeed of participants)
Shares	When a participant shares content from the Smokefree Women page on their own wall
Comments	When a participant comments on a post
Likes	When a participant clicks the “thumbs up” button on a post to indicate “liking”
Comment likes	When a participant “likes” the comment of another participant

#### Phase I

In Phase I of this study, we examined the network structure by conducting social network analysis, which provides a visual and descriptive analysis of the network, including a metric for participant engagement—centrality. We also explore user interactions through automated text analysis, which provides insight on how participant engagement in the network is related to content shared.

##### Data Collection: Full Sample

Data were collected retrospectively in March 2014 from Simply Measured, a social media management marketing platform for all interactions on the Smokefree Women Facebook page, between February 2013 and March 2014. Table 1 provides an overview of Facebook interactions used for this study. All data published on Facebook are publicly available. Personal identifiers were masked to all except members of the research team directly involved in data analysis.

##### Analysis Strategies: Social Network Analysis

Networks consist of nodes and edges where an edge connects 2 nodes, and network structure is determined by the pattern of connectivity between all nodes [13]. In the Smokefree Women social network, page participants are considered to be nodes (dots), and an edge (line) between 2 participants represents that they have interacted during the study period. Interactions consisted of comments on posts unless otherwise noted.

Centrality measures are used to evaluate which of the nodes in a network are most important to the network [13]. As network structure is determined using comments and posts, for the purposes of this study, centrality is also a measure of participant engagement in the network. Eigenvector centrality is the measure of centrality used in this study due to the exploratory nature of the research questions. Eigenvector centrality was calculated for each participant in the study.

Eigenvector centrality is a measure of centrality that is based on a recursive definition where a node’s importance is determined by the importance of adjacent nodes. This is equivalent to evaluating the leading eigenvector of the adjacency matrix. For instance, consider 2 nodes—A and B—that are each connected to 2 additional nodes. Suppose node A is connected to 2 nodes that are connected to several other nodes and thus link 2 communities, whereas node B is connected to 2 nodes that are at the periphery of a network and not connected to any other nodes; node A will have high eigenvector centrality, whereas node B will have low eigenvector centrality.

##### Visualization of the Network

For the sake of simplicity, for all visualizations, the network was treated as an undirected graph where edges (connections between nodes) of the network do not have a direction and only indicate that 2 individuals have interacted. Moreover, the network was treated as unweighted (unless otherwise noted), meaning that an edge can represent 1 interaction with a person or 10 interactions with the same person. This unweighted approach focuses on interpretation of the breadth of interactions between people in the network, as opposed to depth of interactions between any 2 people.

The network was visualized with the Smokefree Women Facebook moderator, both included and excluded. Therefore, when the moderator was not present, the visualization represented participant-to-participant interactions specifically. Moreover, there is a magnification of the network without the presence of the moderator and highlighted centrality with a blue-to-red increasing color scale, where blue nodes are the least central, and red nodes are the most central.

A force-directed layout algorithm was used to position the nodes in the network for the visualizations. Specifically, Fruchterman-Reingold’s [14] algorithm, based on physical forces, was used. Nodes are attracted or repelled based on the connectivity of the network in a way that produces a visually appealing representation [14]. For the purpose of this visualization only, the edges of the graph were weighted to increase the interpretability of the graph. The weights between the edges of the network were set to be the number of interactions between the 2 participants represented by the nodes.

All statistical analyses were conducted with the open-source computing tool, Python version 2.7.5, specifically using SciPy, package version 0.12.0 for plotting and positioning of the force-directed algorithm and NetWorx version 1.7 package for centrality calculations.

##### Analysis Strategies: Automated Text Analysis

Automated text analysis was used to see if a difference existed in topics discussed by participants who are highly engaged and those who are not highly engaged in the network. To identify hubs in the network (ie, participants who have the highest centrality) and characterize the content they contribute compared with other participants in the network, ordered centrality values were plotted, and a threshold for determining the 2 groups (central and peripheral) was chosen to be near the elbow of this curve by visual inspection. To conduct automated analysis of content posted by participants, all participants in the network were divided into 2 groups, split according to the threshold. Subsequently, the top 30 terms preferentially used by those in each group were identified.

To determine the propensity of a term to be used by one group versus the other, a ratio of smoothed relative frequencies was used. Although the moderator was included in the network for calculating the centrality scores, the moderator was excluded from the text analysis to focus on the language that participants use themselves.

#### Phase II

In Phase II of this study, mixed methods were used to further explore participant interactions on the site. For a randomly sampled subset of the population, qualitative content analysis was used to identify salient themes being discussed and self-reported cessation length. Furthermore, correlational analysis was conducted to determine how participant engagement, as measured by centrality, was associated with self-reported cessation length.

##### Data Collection: Subsample

A random sequence generator was used to identify a uniform random sample of 500 participants who interacted on the Smokefree Women Facebook page during the study period. Participants included in this subsample are also included in Phase I of the study. However, in Phase II of the study, qualitative content analysis is conducted to gather more detailed information about information shared by these participants. The sample size of 500 participants was chosen because the size of the dataset was feasible for manual coding, yet likely robust enough to provide a representation of participants in the network.

##### Analysis Strategies: Qualitative Content Analysis

Applied thematic analysis was conducted using an inductive methodology described by Guest et al [15]. Researchers first independently reviewed a subset of the data for familiarization and then reviewed a second time to inductively identify salient themes. These themes were then cross-referenced with previous content analyses of similar topics in an effort to use consistent terms (eg, Burri et al [16]). Once themes were finalized, 2 coders independently analyzed data for 125 of 500 participants (25%) in the subsample to assess inter-rater reliability. An inter-rater reliability of at least .8 agreement using Cohen’s kappa is considered ‘good’. Once the inter-rater reliability threshold of .8 was reached, the remaining sample (n=375) was split, and participant data were coded by 1 of the 2 coders. One coder conducted an additional analysis assessing each post from all 500 participants to extract any self-report of cessation length (measured in days).

##### Analysis Strategies: Correlation Analysis

Correlation between self-reported cessation length and the centrality of participants to the network was analyzed to determine the relationship between Facebook interactions and cessation behaviors. During the qualitative data analysis in Phase II, the longest self-reported cessation length for each of the 500 participants in the subsample was identified. One coder went through all the posts of the subsample of 500 participants and documented any posts reporting cessation length. If a participant reported cessation length in more than 1 post, the longest self-reported cessation length was used in this analysis.

Spearman rank correlation was calculated between centrality in the network and longest self-reported cessation length. Spearman rank correlation was used due to the nonlinear nature of the data. Statistical significance of the correlation was evaluated with no correlation as the null hypothesis.

To evaluate how those actively in the process of quitting compare with those who have been smoke free for some time, a subgroup analysis was performed to evaluate the aforementioned correlation for participants who report longest cessation length of less than 1 year and those who report longest cessation length of more than 1 year.

## Results

Between February 2013 and March 2014, there were 875 posts and 4088 comments from participants on the Smokefree Women Facebook Page and 1166 posts from the moderator. Roughly 4243 people interacted on the page through posts and comments during the 13-month period of data collection. It is of note that participants who interacted on the page did not have to be fans of the Smokefree Women Facebook Page, and thus interactions observed may have been drawn from outside of the 22,000 fans of the page. Additional information about total and average participant interactions (posts and comments) for the entire time period is provided in Table 2.

**Table 2:** Summary of participant Facebook interactions (n=4243).

Action	Participants taking action at least once	Average per participant
Posts	875	2.23
Comments	4088	3.59

### Phase I

#### Social Network Analysis

**Figure 1:**
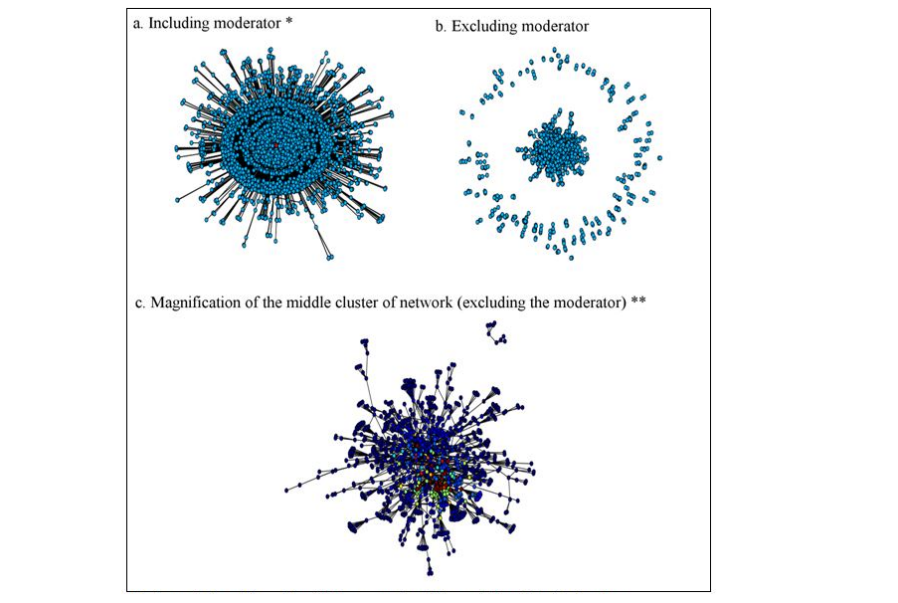
Visualizations of social network. *Moderator indicated by red dot. **Most engaged participants indicated by bright colors.

Figure 1 displays the network structure with and without the moderator. Each participant is indicated by a node, and Facebook interactions between participants are indicated by edges (lines). The moderator is indicated by the red dot in the center of the visualization of Figure 1 A because the moderator is the most engaged participant in the network. The moderator is visibly the connector of every person in the network. In Figure 1 B, absence of the moderator indicates that there is a large, distinct cluster of participants who interact with many other participants in the middle of the network visualization. A separate ring of participants who interact with few others forms around the middle cluster.

Figure 1 C is a magnified visualization of the cluster of participants in the center of the network without the presence of the moderator. Centrality is illustrated with a blue-to-red increasing color scale. Blue nodes are the least central, and red nodes are the most central. There are several highly engaged people who serve as hubs or large connectors, even without the presence of the moderator, as indicated by the bright colors near the center of the network visualization.

#### Automated Text Analysis

A plot of the ordered centralities revealed a threshold for centrality of 0.025 (centrality range: 1×10−7 to 0.7; centrality mean: 0.004). Approximately 100 participants were above this threshold, and 4129 fell below; thus, these participants were labeled as high- and low-engagement participants. Automated text analysis of topics discussed by hubs in the network, or participants with highest centrality compared with other less-engaged participants revealed that hubs, who are most connected to other participants, used more terms of encouragement and congratulations, whereas less-engaged participants discussed more issues related to seeking help, smoking status, and strategies for cessation. The top 30 ranked terms for each group are presented in Table 3.

**Table 3:** Terms used by more-engaged participants and less-engaged participants.

Rank	More-engaged participant terms	Less-engaged participant terms
1	smokefree	Years
2	good	Quit
3	congrats	smoke
4	go	smoking
5	far	Two
6	saved	Year
7	stay	Help
8	keep	I’m
9	great	cigarette
10	strong	since
11	positive	Need
12	well	Cold
13	hang	turkey
14	wtg^a^	Ago
15	yes	Free
16	today	proud
17	get	days
18	don’t	stop
19	water	cigarettes
20	way	smoked
21	think	nicotine
22	better	God
23	awesome	trying
24	come	started
25	job	chantix
26	take	pack
27	cravings	electronic
28	weeks	th^b^
29	wow	Like
30	deep	months

^a^wtg is an acronym for “way to go.”

^b^th likely indicates an ordinal number suffix (eg, 5th, 6th, 7th).

### Phase II

#### Content Analysis

Qualitative analysis of content posted by a subset of 500 randomly sampled participants yielded 1698 unique posts or comments from those participants. Table 4 includes the full list of codes. The most frequently occurring themes of posts and comments were providing support (42.52%, 721 of 1698), announcing number of days smoke free (40.58%,689 of 1698), and giving detailed advice (14.61%,248 of 1698; Table 4). The overall tone of conversation was positive (85.32%, n=1447 of 1698).

**Table 4:** Codes, definitions, and frequencies.

Category	Code	Definition/example	n (%) of Messages (of 1,698)
**Message type**	Post	Post directly on SFW^a^ Facebook wall	165 (9.72)
	Comment	Comment on an existing post on SFW Facebook wall	1533 (90.28)
**Tone toward smoking cessation**	Positive	The emotional tone or sentiment of the message	1447 (85.32)
	Neutral		229 (13.50)
	Negative		20 (1.18)
**Core content**	Providing Support	For example, “You can do it!”	721 (42.52)
	Giving Advice	For example, “Try this…”	248 (14.61)
	Seeking Help	For example, “I can't quit, I need ideas.”	83 (4.89)
	Declaration of Days Smokefree (announce quit date)	For example, “I have been smokefree xx days!”	689 (40.58)
	Relapse	For example, “I broke down yesterday and had a cigarette”	81 (4.77)
	Return from Relapse	For example, “I went back to smoking but I am back and ready to quit again”	67 (3.95)

^a^SFW: Smokefree Women.

#### Correlation Analysis

Of the random subsample of 500 participants, 243 people reported how long they had stopped smoking. The longest reported period of cessation was 35 years, and the shortest period was 1 day. Seven participants reported smoking cessation of exactly 1 year, and median time of smoking cessation was 5 months. There is a significant inverse correlation between cessation length and centrality at the 0.05 level (Spearman correlation coefficient = −0.14, *P*=.03, N=243), meaning that participants who reported longer cessation lengths were less engaged.

**Figure 2:**
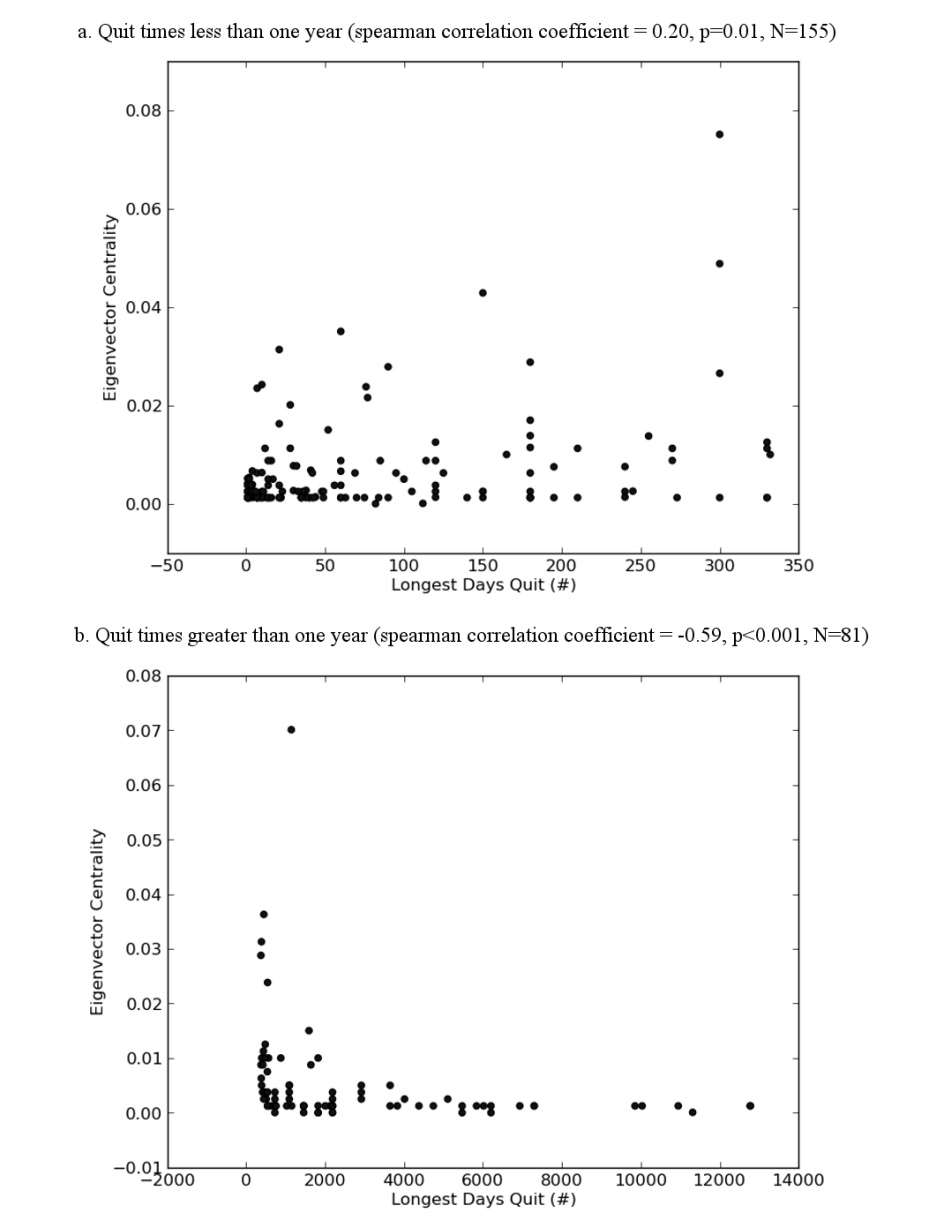
Cessation Length Versus Eigenvector Centrality.

However, splitting the population into those who have been smoke free for less than 1 year versus those who have been smoke free for more than 1 year demonstrates a positive correlation for those who have been smoke free for less than 1 year (Spearman correlation coefficient = 0.20, *P*=.01, N=155) and a strong inverse correlation for those who have been smoke free for more than 1 year (Spearman correlation coefficient = −0.59, *P*<.001, N=81; Figure 2).

## Discussion

### Principal Findings

Network visualization with and without the moderator indicates that participants interact with each other in many small clusters linked by a series of hubs or highly engaged participants (see Figure 1). This suggests that the network is robust to random attack (ie, loss of a participant without regard to their position in the network) but sensitive to selective attack (ie, loss of specific members who are hubs of the network). For example, it is clear that the network is affected quite severely by the loss of the moderator, a particularly important hub of the network [16].

This does not come as a surprise, as participant interaction on a Facebook page is driven, in part, by moderator posts. It is of note that the Facebook platform uses a proprietary algorithm to serve page moderator posts to Facebook participants who have previously interacted with the page. These posts appear in a participant’s home newsfeed, where most interactions on Facebook take place. The more interaction (eg, comments, likes, shares) a moderator post receives, the more Facebook participants are likely to see the post in their newsfeed [11].

Although the moderator serves as a connector of each person in the network, there are several hubs in the network that serve as large connectors of other participants that are less engaged. This finding supports existing evidence for “super participants” in social network sites [17,18]. Having these highly connected super participants is an advantage for the Smokefree Women Facebook page because they help maintain participant-to-participant interaction, rather than only moderator-to-participant interaction. Furthermore, because there are many hubs, when certain hubs leave the network, there are others who continue interacting and connecting participants.

Automated text assessment of content in the network supports findings from the qualitative content analysis in this study, indicating that participants primarily come to the network to provide and receive support and advice, as well as to mark milestones in their smoke-free journey. When stratified by centrality, automated text analysis suggests that highly engaged hub participants use language that is more congratulatory and supportive, whereas other less central participants seek support, discuss strategies for cessation, and announce their smoke-free status.

These findings are further enhanced by findings from the correlational analysis of self-reported smoke-free status, which suggest that participants become more central to the network as they maintain their smoke-free status and use the network for social support but become less involved in the network as maintaining their smoke-free status becomes less difficult and requires less community support. This is consistent with previous research, which found that social network site participants who had recently stopped smoking were more likely to be the first to respond to posts [19]. It is possible that those who have quit more recently feel connected to challenges of other community members and hence assume a more central role.

Taken together, findings from this study suggest that participants who are less central, or are not hubs, are a combination of people at the beginning of their smoke-free journey and people who have been smoke-free for an extended time and only come back to the network to announce their sustained smoke-free status. On contrary, participants who are more central and connect many people (ie, hubs) appear to be those who are further along in their cessation journey and come to the network to provide support and perhaps in the process gain support.

### Limitations

Limitations of this research include lack of analysis of participant demographics; lack of analysis of shares and likes for the study period; and use of self-report for cessation status. This study did not analyze participant demographic information due to privacy restrictions of Facebook that prevented this information from being publicly available. However, as this analysis was focused on properties of the network, demographic details were not central to the goals of this study.

In addition, the study did not include data on shares and likes, additional actions that could be taken by participants in the network, because these data were not available in an automated fashion from Facebook. In a separate analysis, share and like data were manually collected for a period that spanned 3 months. Network visualizations conducted in Phase I were replicated for these 3 months, using all possible Facebook interactions (ie, posts, comments, shares, likes, comment likes). No observable differences were identified when comparing network visualizations for the full sample using only posts and comments as participant interactions, with the subset using all possible Facebook interactions (data not shown).

Moreover, smoke-free status was self-reported by participants on their own volition, sometimes in response to comments on moderator posts asking how long they have been smoke free and at other times in general conversation. Given that the status of everyone in the network is not known, the possibility exists that the number of people who have made a cessation attempt while engaged with the network is underestimated in this study. Furthermore, the correlation analysis may be biased because it did not take into account potential relapse. Nonetheless, it is possible to gain insight on how many participants have made at least 1 cessation attempt through the subset analysis. Future research should explore opportunities to obtain this information from the entire study population and examine the association between cessation length and engagement prospectively.

### Implications for Research and Practice

Ultimately, researchers of social network sites for health seek to understand whether participation in a social network site such as the Smokefree Women Facebook page can lead to better health outcomes (eg, increased quit rates). Although this study was not designed to answer that question, through observation of naturally occurring interactions of the social network and use of methods such as social network analysis, study findings provide insight into the network structure of the social networking site that stand to inform research and practice. Future research may seek to integrate social analysis data with survey data on use of smoking cessation–related social media sites and smoking status or cessation length data to determine the mechanisms by which social media sites can facilitate quit attempts and sustained cessation.

Practitioners may use findings from this study to improve design, implementation, and program evaluation of social network sites focused on health behavior. On the basis of the findings from this study, practitioners may consider the following: (1) developing personas that mimic participants at various points of the cessation trajectory and tailoring the experience with the social network site to fit characteristics of each persona; (2) assessing the best strategies for moderation of the network to determine whether moderator posts that attract comments such as questions, requests for advice, and direct quotes are more effective in providing social support to participants than moderator posts that solicit likes and shares, given the large role the moderator plays in the network; (3) determining whether participants benefit from interaction with only other participants on the Facebook page, or if they also receive benefit from participants in their personal network that may not be participants in that particular social network site for health behavior; and (4) exploring the use of paid advertising on Facebook to boost posts to ensure that users who are new to the community and most in need of support see the posts that will benefit them most.
